# KRAS regulates IL-17 signal activity by affect the metastasis of osteosarcoma via an IL-17A-dependent manner

**DOI:** 10.1093/jbmrpl/ziaf056

**Published:** 2025-04-07

**Authors:** Xing Bao, Na Zhang, Chenchen Wang, Shiwang Liu, Aiqi Fan, Fang Zheng, Caihong Yang

**Affiliations:** Department of Orthopedics, Tongji Hospital of Tongji Medical College, Huazhong University of Science and Technology, Wuhan 430030, People’s Republic of China; Department of Immunology, School of Basic Medicine, Tongji Medical College, Huazhong University of Science and Technology, Wuhan 430030, People’s Republic of China; Department of Immunology, School of Basic Medicine, Tongji Medical College, Huazhong University of Science and Technology, Wuhan 430030, People’s Republic of China; Department of Immunology, School of Basic Medicine, Tongji Medical College, Huazhong University of Science and Technology, Wuhan 430030, People’s Republic of China; Department of Immunology, School of Medicine, Yangtze University, Wuhan 434000, People’s Republic of China; Department of Immunology, School of Basic Medicine, Tongji Medical College, Huazhong University of Science and Technology, Wuhan 430030, People’s Republic of China; Department of Orthopedics, Tongji Hospital of Tongji Medical College, Huazhong University of Science and Technology, Wuhan 430030, People’s Republic of China

**Keywords:** osteosarcoma, KRAS, IL-17A, metastasis, MMPs

## Abstract

Dysregulation of Kirsten rat sarcoma virus (KRAS) plays crucial roles in many tumors. It is reported that KRAS could promote proliferation of osteosarcoma (OS) cells. Nonetheless, the contribution of KRAS to the invasion and spread of OS is still not well understood. This study aimed to investigate KRAS-driven metastasis and the mechanisms behind it in human OS. Tissue microarrays were utilized to assess KRAS expression and its relationship with clinicopathological characteristics. The migratory and invasive abilities of OS cells were evaluated through wound-healing assays and transwell analysis. Furthermore, the regulatory mechanisms of KRAS in human OS were analyzed using RNA sequencing, tandem mass tags assays, multiple immunofluorescence assays, micro-CT, and bioluminescence imaging. In vivo experiments were conducted using established lung metastatic models. Our data showed that downregulation of KRAS in human OS cells could inhibit cell migration and invasion in vitro and in vivo (tumor metastasis model by tail vein injection in BALB/c nude mice). We identify IL-17A, a crucial marker of IL-17 signal pathway, as a downstream target of KRAS. The reduction of KRAS may suppress matrix metalloproteinase (MMP1), MMP3, and MMP9, which are recognized as proteins associated with tumor metastasis, through a mechanism dependent on IL-17 signaling. In summary, these findings indicate that KRAS could serve as a potential biomarker for therapeutic strategies in human OS. Mechanistically, our data revealed that KRAS knockdown affects tumor metastasis by inactivating IL-17 signal pathway via IL-17A-dependent manner.

## Introduction

Osteosarcoma (OS) represents the most common primary malignant tumor of the bone. While there have been advancements in the survival rates for OS in recent years, the overall cure rate for patients remains low, largely because of the tumor’s aggressive nature and swift progression to distant metastases.^1–3^ Osteosarcoma metastasis appears most commonly in the lung and is the prime cause of death for patients with OS. Identifying the primary factors that may influence the aggressive biological behavior of OS, particularly concerning metastasis, is essential. The Kirsten rat sarcoma virus (KRAS) gene, part of the rat sarcoma virus (RAS) gene family, is known to be involved in tumor development. Research conducted in the past has established a link between KRAS and OS, suggesting that KRAS may enhance the proliferation of OS cells.^4^^,^^5^ Kirsten rat sarcoma virus could promote gastric tumorigenesis and metastasis in a genetically engineered mouse model.^6^ Nonetheless, there is limited understanding regarding the involvement of KRAS in the processes of invasion and metastasis in OS. Kirsten rat sarcoma virus is notorious undruggable target, which is difficult to be effectively targeted by traditional small molecule drugs. Current thinking is that the KRAS work together with p53 to promote tumor progression.^7^ As a result, concentrating on the downstream molecules associated with the KRAS gene is of particular significance.

IL-17 signaling has been demonstrated to promote the development of various tumors.^8^ As a crucial component of the IL-17 signaling pathway, IL-17A serves as an immune and inflammatory mediator exhibiting diverse biological functions.^9^ It is commonly found within the inflammatory microenvironment of numerous tumors and plays a role in enhancing the migration and invasion of tumor cells, as well as contributing to chemotherapy resistance and immunosuppression, which ultimately results in tumor progression and metastasis. Clinical studies indicate that elevated levels of IL-17A act as an independent predictor of unfavorable survival outcomes in cancer patients.^10–13^ Kirsten rat sarcoma virus has the potential to trigger IL-17 signaling in PanIN (pancreatic intraepithelial neoplasia) and promote the infiltration of IL-17-producing immune cells into the pancreatic stroma.^14^ However, it remains unclear whether this mechanism involving KRAS and IL-17 signaling pathways are also present in OS.

The present study aims to investigate KRAS-mediated metastasis and the underlying mechanisms in human OS. In this research, the inhibition of KRAS expression in human OS cells has the potential to reduce both cell migration and invasion in vitro as well as in vivo (Metastatic capabilities of OS cells were assessed after the intravenous injection of cells into the tail vein of mice). Our findings indicate that the knockdown of KRAS influences tumor metastasis through the inactivation of the IL-17 signaling pathway. We identify IL-17A, a crucial marker of IL-17 signal pathway, as a downstream target of KRAS. Mechanistically, our data reveal that knockdown of KRAS inhibits matrix metalloproteinase (MMP1), MMP3, and MMP9, which are known as tumor metastasis-related proteins, via an IL-17 signal-dependent manner. Collectively, these findings imply that KRAS may serve as a promising biomarker for therapeutic interventions in human OS.

## Materials and methods

### Clinical specimens

A total of 86 OS tumor samples, all with complete follow-up, were gathered in accordance with protocols sanctioned by the ethics committee of Tongji Hospital. Prior to surgery, no patients underwent any antitumor therapies. Informed consent, prepared in line with ethical guidelines, was secured from each patient. The clinical characteristics of these individuals can be found in Table 1. Fresh tissue samples were preserved in liquid nitrogen prior to RNA extraction. Clinical and histopathological data were documented through a retrospective examination of patient records.

**Table 1:** The relationship between KRAS expression and clinicopathological variables of OS.

Items	KRAS		*p*-value
	Low	High	
**All cases**	43	43	
**Age (year)**			.8294
** ≥18**	23	21	
** <18**	20	22	
**Gender**			.8283
** Male**	25	23	
** Female**	18	20	
**Anatomical location**			.6610
** Limb bone**	24	27	
** Axial bone**	19	16	
**Grade of tumor**			.0023
** Low**	28	13	
** High**	15	30	

### Cell culture

Human OS cell lines, namely MG-63, KHOS, U-2OS, and Saos-2, were sourced from the American Type Culture Collection (ATCC). The cell lines utilized in this research have undergone authentication through STR analysis by Beijing Microread Genetics Co., Ltd. The MG-63 and KHOS cell lines were grown in RPMI 1640 medium (HyClone, SH30027.01) supplemented with 10% fetal bovine serum (FBS, Gibco, 10100147), whereas U-2OS and Saos-2 cells were maintained in Dulbecco’s modified Eagle’s medium (DMEM, HyClone) with the addition of 10% FBS. All cultures were kept at 37 °C in a 5% CO_2_ environment.

### Transfection

KRAS stably knockdown and IL-17A stably expressed OS cells (KHOS and Saos-2 cell lines) were infected with the lentivirus, synthesized by Shanghai Genechem Co., Ltd, and selected with puromycin (1 mg/mL) for 4 wk.^15^ Transfection efficiency of KRAS knockdown lentivirus (GFP labeled) was detected in flow cytometry (FCM) experiments and Immunofluorescence assay. High GFP expression suggests high transfection efficiency.

### Transwell and Matrigel invasion assays

A total of 6 × 10^4^ OS cells (KHOS and Saos-2 cells with KRAS knockdown and IL-17A upregulated) were seeded into the upper chambers of noncoated (3422, Corning) or Matrigel-coated (354480, Corning) Transwell plates with membranes containing 8.0-μm pores. At the conclusion of the culture period, remove the chamber and discard the medium from the upper chamber. Gently rinse the chamber with PBS to eliminate any residual medium and wipe away any cells that have not migrated or invaded the upper chamber. Subsequently, place the chamber in a container filled with fixative solution for fixation, followed by staining with crystal violet. The average numbers of cells were calculated from at least 6 fields by 2 independent researchers.

### Wound-healing assay

Stably transfected KHOS and Saos-2 cells were seeded in 6-well plates and cultured in different CM (Complete Medium) for 48 h. Once the confluency attained 80%, a straight artificial wound was created using a 200-μL pipette tip. The cells were then cultured in a serum-free medium. Images were acquired at 0, 12, and 24 h using a phase-contrast microscope (Leica, Leica Microsystems), and the analysis of the images was carried out using Image-Pro Plus software.

### RNA-sequencing

RNA was extracted from KHOS cells that were transfected, utilizing TRIzol reagent (15596018, Invitrogen). Subsequent steps, including RNA sample quantification, qualification, library preparation, and RNA sequencing (RNA-seq), were carried out by Novogene Co., Ltd. The edgeR R package (3.12.1) was employed to conduct differential expression analysis between the 2 conditions. For defining significantly different expression, a threshold of corrected *p*-values at .05 and absolute fold-changes of 2 was established.^16^ This process was repeated in 3 independent experiments.

### Tandem mass tags analysis

Tandem mass tags (TMT) for KHOS cells was performed and analyzed by Novogene Co., Ltd as previously described.^17^ Each protein sample was taken, and the volume was made up to 100 μL with DB (Dilution Buffer) dissolution buffer. Trypsin and a 100 mM TEAB buffer were introduced to the sample, which was then thoroughly mixed and incubated at 37 °C for 4 h. Following this, trypsin and CaCl_2_ were incorporated, allowing the sample to undergo digestion overnight. Formic acid was combined with the digested sample, the pH was adjusted to below 3, and then centrifugation was performed at 12 000 *g* for 5 min at room temperature. The resulting supernatant was carefully transferred to a C18 desalting column and washed 3 times using a washing buffer (0.1% formic acid, 3% acetonitrile). Subsequently, elution was carried out with an elution buffer (0.1% formic acid, 70% acetonitrile). The eluates from each sample were collected and subjected to lyophilization. To reconstitute the samples, 100 μL of 0.1 M TEAB buffer was added, followed by 41 μL of a TMT labeling reagent dissolved in acetonitrile, and the mixture was shaken for 2 h at room temperature. The reaction was thereafter halted by the addition of 8% ammonia. All labeled samples were then combined in equal volumes, desalted, and freeze-dried. Peptides were separated in a homemade analytical column and subjected to LC-mass spectrometry (MS)/MS analysis. A fold change (FC) > 2 or < 0.5 with a *p* < .05 was considered as significance for differential expression. Three independent experiments were performed.

### Western blot analysis

Equal amounts of proteins collected from different kinds of cell lysates (described in Cell culture and Transfection sections) were loaded on 10%-15% SDS-PAGE gels using a NuPAGE system (Invitrogen) and then transferred onto PVDF membranes as previously described.^18^ Western blot was performed with rabbit polyclonal anti-KRAS, anti-IL-17A, anti-CEBPB, anti-MMP1, anti-MMP3 and anti-MMP9 antibodies (1:100 dilution). Sections stained with nonimmune rabbit serum (1:100 dilution) in phosphate-buffered saline (PBS) instead of primary antibody served as negative controls. Anti- KRAS (12063-1), anti-IL-17A (66148-1) were from Proteintech. Anti-CEBPB (43095), anti-GAPDH (5174S), and anti-MMP1 (54376) were from Cell Signaling Technology. Anti-MMP3 (ab52915) and anti-MMP9 (ab283575) were from Abcam.

### Quantitative RT-PCR

Total RNA was isolated utilizing Trizol reagent (Invitrogen). cDNA synthesis was conducted with a RevertAidTM First Strand cDNA Synthesis kit (Fermentas), and real-time quantitative PCR was performed using the SYBR-Green PCR Master Mix (Applied Biosystems) on a 7900 Real-Time PCR System (Applied Biosystems). Endogenous controls, including U6 or GAPDH, were employed. The amplification reactions took place in a 96-well optical plate (Applied Biosystems) at a temperature of 94 °C for 2 min, followed by 38 cycles consisting of 94 °C for 45 s, 56 °C for 45 s, and 72 °C for 40 s.

### FCM experiments

Cells were fixed in 70% ethanol and digested with RNase A. Transfected cells with GFP labeled were analyzed with Annexin V/FITC kit (BD Biosciences).

GFP labeled cells were quantified using FCM according to the manufacturer’s instructions.^19^ Fix the cells using 4% paraformaldehyde for 15 min at room temperature. Following fixation, wash the cells with PBS and then permeabilize them using a permeabilization buffer (such as 0.1% Triton X-100 in PBS) for 10-15 min at room temperature. Subsequently, add the fluorescent-conjugated antibodies targeting the intracellular antigens and incubate in the dark at 4 °C for 30 min. BD Accuri C6 Flow Cytometer was used for this experiment.

### Immunohistochemistry and immunofluorescence assay

Immunohistochemistry (IHC) staining was performed as previously described.^20^ Paraffin sections were reacted with rabbit polyclonal anti-KRAS, anti-IL-17A, anti-CEBPB, anti-MMP1, anti-MMP3 and anti-MMP9 antibodies (1:100 dilution). Sections stained with non-immune rabbit serum (1:200 dilution) in phosphate-buffered saline (PBS) instead of primary antibody served as negative controls. Anti-KRAS (12063-1) and anti-IL-17A (66148-1) were from Proteintech. Anti-CEBPB (43095) and anti-MMP1 (54376) were from Cell Signaling Technology. Anti-MMP3 (ab52915) and anti-MMP9 (ab283575) were from Abcam. Cells with positive staining were enumerated in a minimum of 12 representative fields, and the mean percentage of positively stained cells was determined. The evaluation of immunostaining was performed by 2 independent pathologists who were blinded to the clinical characteristics and outcomes.

In the immunofluorescence assay, fixed cells were treated with 0.1% Triton X-100 at room temperature for 15 min to permeabilize them, followed by overnight incubation with the primary antibody at 4 °C. After washing the cells 3 times with PBST (phosphate-buffered saline with Tween-20), they were incubated at room temperature for 1 h with the Alexa Fluor 488 conjugated goat anti-rabbit secondary antibody.^20^ Nuclei were stained with PBS with 2 μg/mL DAPI for 4 min. The cells were then analyzed using confocal microscopy (FV10i, Olympus).

### Enzyme-linked immunosorbent assay

We performed centrifugation of the serum from nude mice and the cell supernatants at a force of 1000 *g* for 15 min at a temperature of 4 °C, subsequently collecting the resulting supernatants for analysis. To quantify the relative concentrations of IL-17A in the samples, we utilized ELISA kits for IL-17A (human, KE00203, Proteintech) and IL-17A (mouse, KE10020, Proteintech).

### Tumor xenografts

Female BALB/c nude mice were obtained from the Experimental Animal Center at Vitalriver Company and kept under specific pathogen-free conditions. All procedures for animal care and management complied with the guidelines set forth by the National Institutes of Health for the care and use of laboratory animals. The metastatic potential of KHOS cells (5 × 10^6^ cells) was evaluated after injecting the cells intravenously into the tail vein of 6-wk-old mice (*n* = 10 per group). Mice with tumors were euthanized at week 6, and their lungs were harvested for subsequent analysis.

### Analysis of tumor metastatic capacity by in vivo bioluminescence imaging

Bioluminescence imaging was performed according to the manufacturer’s instructions.^21^ The mice were placed under anesthesia using 2% isoflurane and were given an intraperitoneal injection of D-luciferin (80 μL; 40 mg/mL; Biotium) 10 min before imaging, which was conducted while the animals were in a lateral position. An IVIS 200 imaging system along with Living Image software Version 3.0.4 (Xenogen) was utilized for the imaging process. Following a 5-s exposure, the total flux of the ROI was measured as photons per second for each individual animal.

### Statistical analysis

All statistical analyses were conducted using the SPSS19.0 software package alongside GraphPad Prism 9 (GraphPad Software). The Kaplan–Meier survival analysis employed the log-rank test. Results are presented as mean ± SD from a minimum of 3 independent experiments, and statistical assessments were carried out using one-way ANOVA or Student’s *t*-tests. *p*-values of <.05 or .01 were regarded as statistically significant.

## Results

### Knock down of KRAS attenuates the migratory and invasive capacities of OS cells

**Figure 1:**
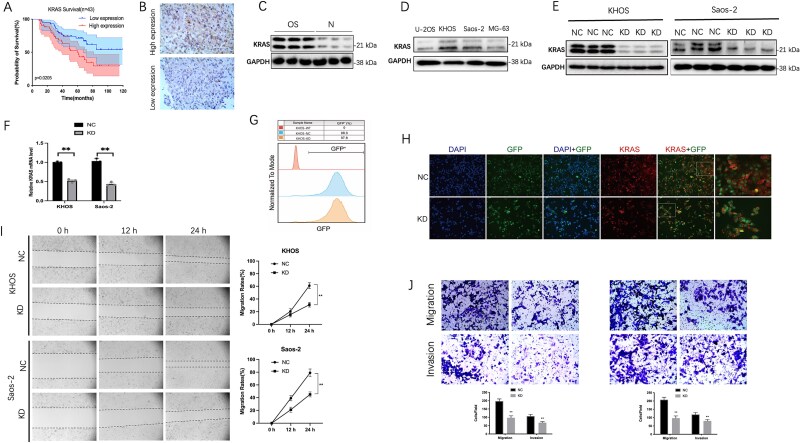
The inhibition of KRAS reduces the migratory and invasive abilities of OS cells. (A) Kaplan–Meier survival analysis indicated that patients exhibiting high levels of KRAS expression had a notably shorter overall survival time compared to those with low KRAS expression (log-rank *p* = .0205, *n* = 43 for each group). (B) Images demonstrating either low or high levels of KRAS expression through IHC staining are presented. (C) Representative western blot indicated that the expression of KRAS was significantly elevated in OS in comparison to normal bone (N). (D) Proteins were examined by western blotting in human OS cell lines U2OS, MG63, Saos2, and KHOS. (E) Western blot assay showed the level of KRAS was sharply reduced in OS cells transfected with lentivirus (KD). (F) qPCR showed a significant decrease in RNA levels. (G) Satisfactory transfection efficiency of KRAS knockdown lentivirus (GFP labeled) was detected in FCM experiments. (H) Immunofluorescence assay showed high efficiency of KRAS knockdown (GFP labeled). (I) The migration of OS cells in wound-healing assays was reduced by the downregulation of KRAS. Migration rates were determined by the ratio of the healing area to the wound area. (J) The knockdown of KRAS notably inhibited both the migration (up) and invasion (down) of OS cells as shown by transwell analysis. The results are expressed as mean ± SD from 3 independent experiments. Statistical analysis was performed using ANOVA or *t*-tests. A significant difference between the groups indicated is marked by ^*^p < .05, ^**^p < .01, or ^***^p < .001.

Previous investigations have shown that KRAS displayed dysregulation in different types of tumors.^7^ Our findings confirmed a negative association between KRAS levels and overall survival rates in human OS. The OS cohort was categorized into 2 groups: high expression (*n* = 43) and low expression (*n* = 43), based on the median relative expression of KRAS mRNA. All patients had not undergone any antitumor treatments prior to surgical intervention. As depicted in Figure 1A, we assessed the relationship between KRAS expression and patient prognosis in OS. The Kaplan–Meier survival analysis revealed that patients exhibiting elevated KRAS expression had a significantly reduced overall survival duration compared to those with lower KRAS levels. The expression levels of the KRAS protein were further assessed by IHC staining. Notably, KRAS protein levels were found to be elevated in patients exhibiting higher KRAS mRNA levels. Images depicting representative high or low KRAS expression levels are presented in Figure 1B. Additionally, a western blot analysis was conducted on 6 samples obtained from both normal bone and OS tissues. The detection of KRAS expression occurred in both tissue types, with a notable increase observed in OS compared to the normal bone (Figure 1C). The expression of KRAS was analyzed in the corresponding cell lines U-2OS, MG-63, Saos-2, and KHOS by western blot. As shown in Figure 1D, KRAS expression was higher in KHOS and Saos-2 cells, which displayed high migratory and invasive capacities in publications.^15^ To explore the effects of KRAS in KHOS and Saos-2 cell lines, we used lentiviral transfection to knock down KRAS, and KRAS downregulation was confirmed by western blot and qPCR (Figure 1E and F). Transfection efficiency of KRAS knockdown lentivirus was detected in FCM experiments and Immunofluorescence assay (Figure 1G and H). Compared with KHOS WT cell line (KHOS-WT), the GFP efficiency was significantly increased in KRAS knockdown KHOS cell line (KRAS-KD) and negative control KHOS cell line (KRAS-NC). Wound healing and transwell assays were performed to explore the effects of KRAS on the migratory and invasive capacities in KHOS and Saos-2 cell lines. As shown in Figure 1I, downregulation of KRAS reduced the migration of OS cells in wound-healing assays. Additionally, transwell assays indicated that the reduction of KRAS expression diminished the invasive capacity of OS cells (Figure 1J). These findings suggest that targeting KRAS can effectively impair the migration and invasion of human OS cells.

### RNA-seq identified differential transcripts in KRAS downregulated OS cells

**Figure 2:**
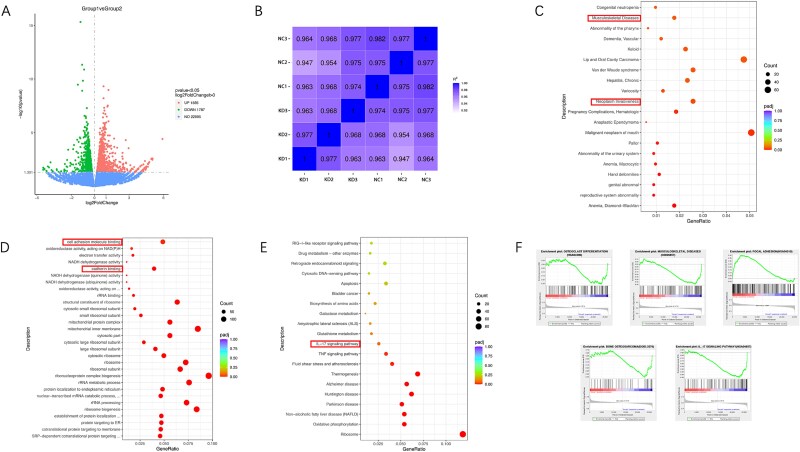
Differential transcripts were identified in OS cells with downregulated KRAS through RNA-seq. (A) Volcano plots compared KRAS knock-down KHOS cells to control samples. The transcripts that were significantly upregulated and downregulated are displayed (|logFC| > 1, *p* < .05, FDR < 0.01). (B) Correlation coefficient plots for each sample. (C) DisGeNET analysis showing the enrichment in musculoskeletal diseases and neoplasm invasiveness. (D) GO analysis demonstrating DEGs involving in the process of cell adhesion molecule binding and cadherin binding, which was indicated by red frame. (E) KEGG pathways analysis revealing the enrichment in IL-17 signaling pathway. (G) GSEA validated enrichment of osteoclast differentiation, musculoskeletal diseases, focal adhesion, bone OS, and IL-17 signaling pathway.

RNA sequencing was conducted on KHOS cells with KRAS downregulation, resulting in changes to transcript expression compared to control cells, including both upregulated and downregulated transcripts (Figure 2A). Correlation coefficient plots for each sample are shown in Figure 2B. DisGeNET analysis showed the enrichment in musculoskeletal diseases and neoplasm invasiveness (Figure 2C). Gene Ontology (GO) analysis demonstrated differentially expressed genes (DEGs) involved in the process of cell adhesion molecule binding and cadherin binding, which was indicated by the red frame (Figure 2D). Kyoto Encyclopedia of Genes and Genomes (KEGG) pathways analysis revealed the enrichment in IL-17 signaling pathway (Figure 2E). Gene set enrichment analysis (GSEA) revealed a significant association between DEGs and processes such as osteoclast differentiation, musculoskeletal disorders, focal adhesion, bone OS, and the IL-17 signaling pathway (Figure 2F).

### TMT labeling coupled with MS detection of altered proteins in KRAS downregulated OS cells

**Figure 3:**
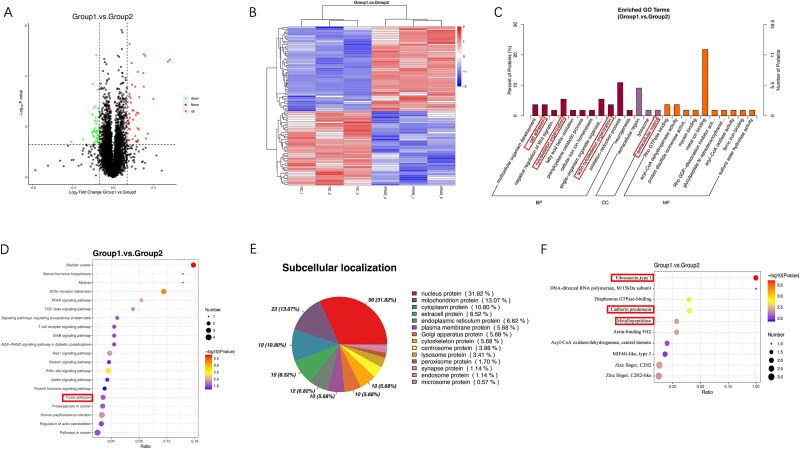
TMT labeling coupled with MS detection of altered proteins in KRAS-KD (Group1) and KRAS-NC (Group2) OS cells. (A) Volcano plot was also used to display altered proteins. (B) Heat map and hierarchical clustering of DEPs in KRAS-KD and KRAS-NC groups. (C) GO enrichment analysis of the proteomic profiles of KRAS-KD and KRAS-NC groups. These proteins were markedly enriched in terms of cell adhesion, cytoskeleton organization, actin cytoskeleton organization and extracellular matrix. (D) KEGG analysis revealing the enrichment in focal adhesion. (E) Subcellular localization for differential proteins. (F) IPR analysis showing the enrichment in fibronectin, cadherin prodomain and metallopeptidase.

To comprehensively analyze the differentially expressed proteins (DEPs) in host cells during KRAS knockdown lentivirus (Lv-shKRAS) infected KHOS cells, TMT labeling coupled with LC-MS/MS analysis was conducted in cells with KRAS knockdown. In total, spectral analysis identified 6485 peptides across 3 distinct biological replicates from 2 groups. Using the specified thresholds of an FC greater than 1.2 or less than 0.83 and a *p*-value of <.05, we identified 92 DEPs, of which 49 were significantly upregulated and 43 downregulated in KRAS-KD cells (Group 1) compared to KRAS-NC cells (Group 2) (Figure 3A and B). To explore the functional roles of these identified DEPs in the KRAS knockdown group, we conducted GO analysis. The results indicated a significant enrichment of these proteins in processes related to cell adhesion, cytoskeleton organization, actin cytoskeleton organization, and extracellular matrix (Figure 3C). Additionally, to explore the comprehensive downstream pathways connected to KRAS, we performed KEGG pathway enrichment analysis. The findings showed that the identified 92 DEPs were primarily enriched in focal adhesion (Figure 3D). Subcellular localization for differential proteins is shown in Figure 3E. IPR analysis showed the enrichment in fibronectin, cadherin prodomain and metallopeptidase (Figure 3F), which play crucial roles in the process of invasion and migration.

### Inhibition of IL-17 signaling mediated the effects of KRAS knockdown on OS cell migration and invasion

**Figure 4:**
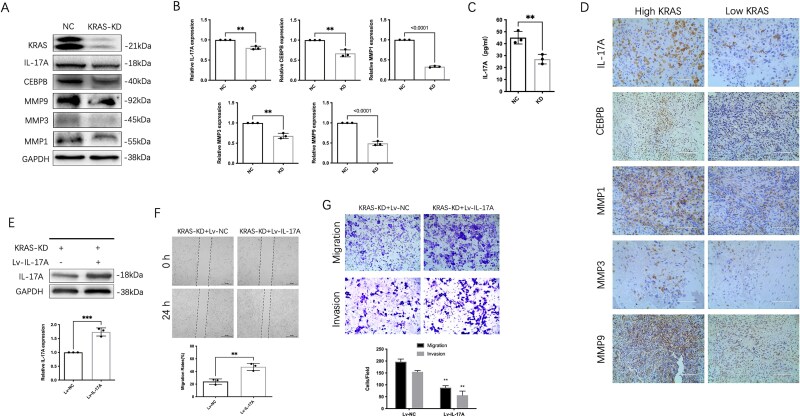
Inhibition of IL-17 signaling mediated the effects of downregulation on OS cell migration and invasion. (A) Western blot showed that downstream targets of IL-17 signaling (CEBPB, MMP1, MMP3, and MMP9) decreased activation in the cells relative to control. (B) qPCR showed a significant decrease in RNA levels. (C) ELISA analysis showed IL-17A levels decreased after KRAS knockdown. (D) Representative images showed markers of IL-17 signaling in high or low expression of KRAS in OS tissues. (E) Western blot assay and qPCR showed the level of IL-17A was sharply increased in OS cells transfected with lentivirus(Lv-IL-17A). (F) Upregulation of IL-17A increased the migration of OS cells in wound-healing assays. (G) The overexpression of IL-17A markedly enhanced the migration (up) and invasion (down) capabilities of OS cells, as assessed by transwell analysis. The data are presented as mean ± SD (*n* = 3). ^**^p < .01 or ^***^p < .001 signifies a statistically significant difference between the specified groups.

Kyoto Encyclopedia of Genes and Genomes pathways analysis revealed the enrichment in IL-17 signaling pathway in KRAS-KD cells. While the IL-17 signaling pathway plays a vital role in metastasis and is generally activated in various tumors,^22^ we supposed that knockdown of KRAS might suppress tumor metastasis through inhibition of IL-17 signaling pathway. The KRAS downregulation cell lines showed decreased expression of IL-17A when compared with the relevant control cell lines (Figure 4A). Immunohistochemical staining showed that IL-17A+ cells were mainly distributed in the tumor tissue, while a few CD45 + immune cells were observed in the same area (Figure S1A). To further verify that IL-17A in OS tissues was secreted by tumor cells, immunofluorescence was used to detect the distribution of the OS cell marker protein SATB2 and IL-17A. We found that IL-17A was expressed by SATB2 + OS cells (Figure S1B). These data suggested that tumor cells are the primary cells that produce IL-17A in OS.

Furthermore, we assessed the expression levels of downstream targets of IL-17 signaling pathway. CEBPB, MMP1, MMP3, and MMP9 showed decreased activation in the cells relative to control (Figure 4A and B). Furthermore, ELISA analysis showed IL-17A levels decreased after KRAS knockdown (Figure 4C). We made a further exploration of the clinical correlation between KRAS and IL-17 signaling pathway in our study. IHC assay of KRAS, IL-17A, CEBPB, MMP1, MMP3, and MMP9 was performed using the human OS tissue microarray. Markedly, IL-17A and downstream target expression positively correlated with KRAS (Figure 4D). To confirm IL-17 signaling mediated the effects of KRAS knockdown on OS cell migration and invasion, IL-17A was overexpressed in KRAS knockdown cells. As shown in Figure 4E, western blot assay and qPCR assay showed the level of IL-17A was sharply increased in OS cells transfected with lentivirus (Lv-IL-17A). Upregulation of IL-17A increased the migration of OS cells in KRAS knockdown cells (Figure 4F). Besides, IL-17A overexpression significantly increased the migration and invasion of OS cells with transwell analysis (Figure 4G). These results suggest that IL-17 signaling mediated the effects of KRAS knockdown on OS cell migration and invasion.

**Table 2:** Lung metastasis rate of KHOS-NC cells and KHOS-KD cells.

Cells	Total (*n*)	Lung metastasis (*n*)	No metastasis (*n*)	Metastasis rate (%)
**KHOS-NC**	10	8	2	80
**KHOS-KD**	10	3	7	30

### IL-17 signaling mediated the inhibition of pulmonary metastasis caused by KRAS knockdown in vivo

**Figure 5:**
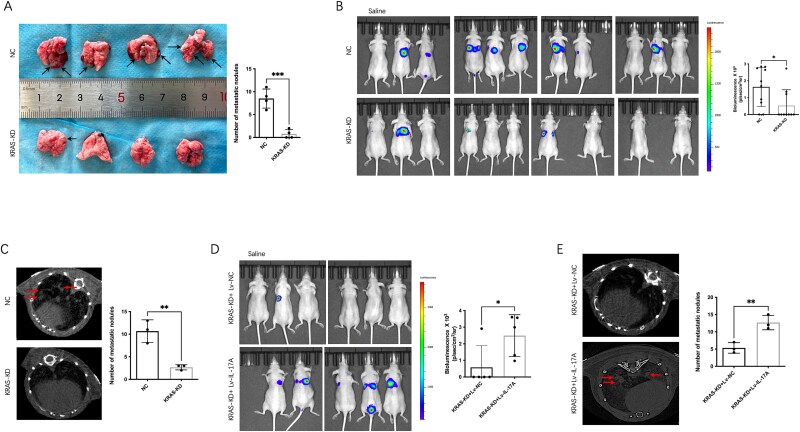
Analysis of lung metastasis formed by KHOS-Luci cells in vivo. (A) Representative images of lungs on day 70 after mice were injected with KHOS-Luci cells (*n* = 4 per group) (metastatic nodules are indicated by black arrows). (B) BLI of tumor-bearing mice (*n* = 10 per group). (C) Micro-CT of metastatic lung nodules formed by stable downregulated KRAS or control KHOS cells is presented. The number of metastatic nodules of the lungs of mice is presented (metastatic nodules are indicated by arrows). (D) BLI showed IL-17A overexpression significantly increased lung metastasis (*n* = 5 per group). (E) Micro-CT showed IL-17A overexpression significantly increased lung metastasis compared to control group. The number of metastatic nodules of the lungs of mice is presented (metastatic nodules are indicated by red arrows). ^*^p < .05, ^**^p < .01 or ^***^p < .001 indicates a significant difference between the indicated groups.

To further investigate the function of KRAS in human OS metastasis, analysis of lung metastasis formed by KHOS-Luci cells was performed in nude mice. These KHOS cells used in vivo are the same as those used in vitro in previous figures. As anticipated, the lung metastasis of the tumor was significantly reduced when KHOS cells with stable KRAS knockdown were implanted (3/10 mice lung metastasis) compared to the control group (8/10 mice lung metastasis, Table 2) (Figure 5A). Bioluminescence imaging and Micro-CT showed the tumor metastatic capacity was inhibited in KRAS knockdown group (Figure 5B and C). While IL-17A overexpression significantly rescued the inhibition of lung metastasis caused by KRAS knockdown (Figure 5D and E).

**Figure 6:**
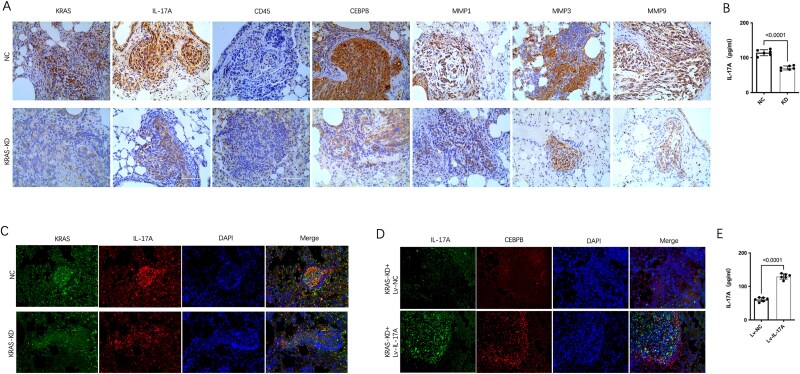
IL-17 signaling mediated the inhibition of pulmonary metastasis caused by KRAS knockdown in vivo. (A) IHC results indicated the differences in the expression of CD45 and IL-17 signaling markers (IL-17A, CEBPB, MMP1, MMP3, MMP9) between the stable KRAS knockdown group and the control group. Representative images were shown at 400× magnification. Scale bars, 100 μm. (B) ELISA assay showed that knockdown of KRAS reduced the serum level of IL-17A in nude mice. (C) Multiple immunofluorescence assay showed IL-17A expression was inhibited in KRAS knockdown group. (D) IL-17A overexpression significantly increased CEBPB in lung metastasis models. Representative images were shown at 200× magnification. Scale bars, 50 μm. (E) ELISA analysis showed that IL-17A upregulation increased the serum level of IL-17A in nude mice.

In addition, IHC outcomes indicated the levels of IL-17A, CEBPB, MMP1, MMP3 and MMP9 were lowered by KRAS knockdown (Figure 6A). In addition, ELISA assay confirmed that knockdown of KRAS reduced the serum level of IL-17A in nude mice (Figure 6B). Multiple immunofluorescence assays showed IL-17A expression was inhibited in KRAS knockdown group (Figure 6C). While upregulation of IL-17A could reverse the inhibition of CEBPB caused by KRAS knockdown in the lung metastasis model (Figure 6D). Further, ELISA analysis showed that overexpression of IL-17A increased the serum level of IL-17A in nude mice (Figure 6E).

The findings indicated that KRAS is significant in the metastasis of OS cells. By suppressing IL-17 signaling, knockdown of KRAS could downregulate MMP1, MMP3 and MMP9, thereby reducing OS cell metastasis.

## Discussion

Osteosarcoma represents the most prevalent primary malignant tumor of the bone. The survival rate for OS patients continues to be quite low due to its aggressive nature and the rapid progression of distant metastases. Research has demonstrated that the dysregulation of KRAS influences both the proliferation and invasion of tumor cells.^23–25^ Nonetheless, the specific roles of KRAS, along with the mechanisms involved in human OS, remain largely unknown. In this study, we discovered that KRAS influences the IL-17 signaling pathway, thereby affecting cell migration and invasion in human OS. The IL-17 signaling pathway is crucial for various biological processes, and its aberrant activation contributes to the development of numerous tumors.^9^ Three RAS genes, known as HRAS, KRAS, and NRAS, have been discovered within the mammalian genome. The KRAS gene is often acknowledged as a homolog of the Kirsten murine sarcoma virus, playing a crucial role in the malignant transformation of rodent cells.^7^ Human KRAS is situated on chromosome 12p12.1 and comprises 6 exons. The KRAS protein is consistently expressed across various tissues, although its expression is significantly elevated in select areas, including skeletal muscle, myocardium, uterus, adrenal cortex, and certain stem cells found in bone marrow.^7^

Bioinformatics analysis in previous studies identified a significant distinction in the KRAS gene at the gene level between samples of metastatic and nonmetastatic OS.^26^ However, further analysis is needed to verify the results. Oncogenic KRAS is the most mutated oncogene in human cancers.^27^ P53 and KRAS are 2 notorious undruggable targets, which are difficult to be effectively targeted by traditional small molecule drugs.^7^ Current thinking is that the KRAS work together with p53 to promote tumor progression. Therefore, it is particularly important to focus on the downstream molecules of KRAS gene.

Studies have revealed that IL-17 is intricately involved in the tumor’s pathophysiology, influencing processes such as tumorigenesis, proliferation, angiogenesis, and metastasis, as well as enabling the tumor to develop resistance to both immune responses and chemotherapy.^28^ It is reported that KRAS could promote proliferation of OS cells. Additionally, another study indicated that KRAS is a target of miRNAs. Previously documented findings suggest that several miRNAs can inhibit OS by directly targeting KRAS, highlighting its significance as an essential miRNA target that regulates OS growth.^29^ Thus, KRAS could be an important miRNA target that modulates the growth of OS. Most of these studies have focused on upstream molecules of KRAS.^30^ However, little is known about the downstream targets of KRAS and the mechanisms in OS. In this study, we focused on the KRAS downstream signaling pathway. According to our study, KEGG pathways analysis revealed the enrichment in IL-17 signaling pathway in KRAS-KD cells. Although the IL-17 signaling pathway is crucial for metastasis and is often activated in different types of tumors, we hypothesized that reducing KRAS expression could hinder tumor metastasis by blocking the IL-17 signaling pathway. The inflammatory response is more significant in tumor tissues with high KRAS expression, and IL-17 signaling pathway plays an important role in the inflammatory response. Aberrant IL-17A activation mediates KRAS-induced inflammation. The KRAS downregulation cell lines showed decreased expression of IL-17A when compared with the relevant control cell lines. This study suggested that tumor cells rather than immune cells are the primary cells that produce IL-17A in OS. Furthermore, the expression levels of downstream targets of IL-17 signaling pathway were assessed. CEBPB, MMP1, MMP3, and MMP9 showed decreased activation in the cells relative to control. Matrix metalloproteinases (MMPs) are known to be involved in various biological processes, especially in tumor invasion and metastasis. MMP1, MMP3, and MMP9 have also been shown to play critical roles in tumor metastasis.^31^

**Figure 7:**
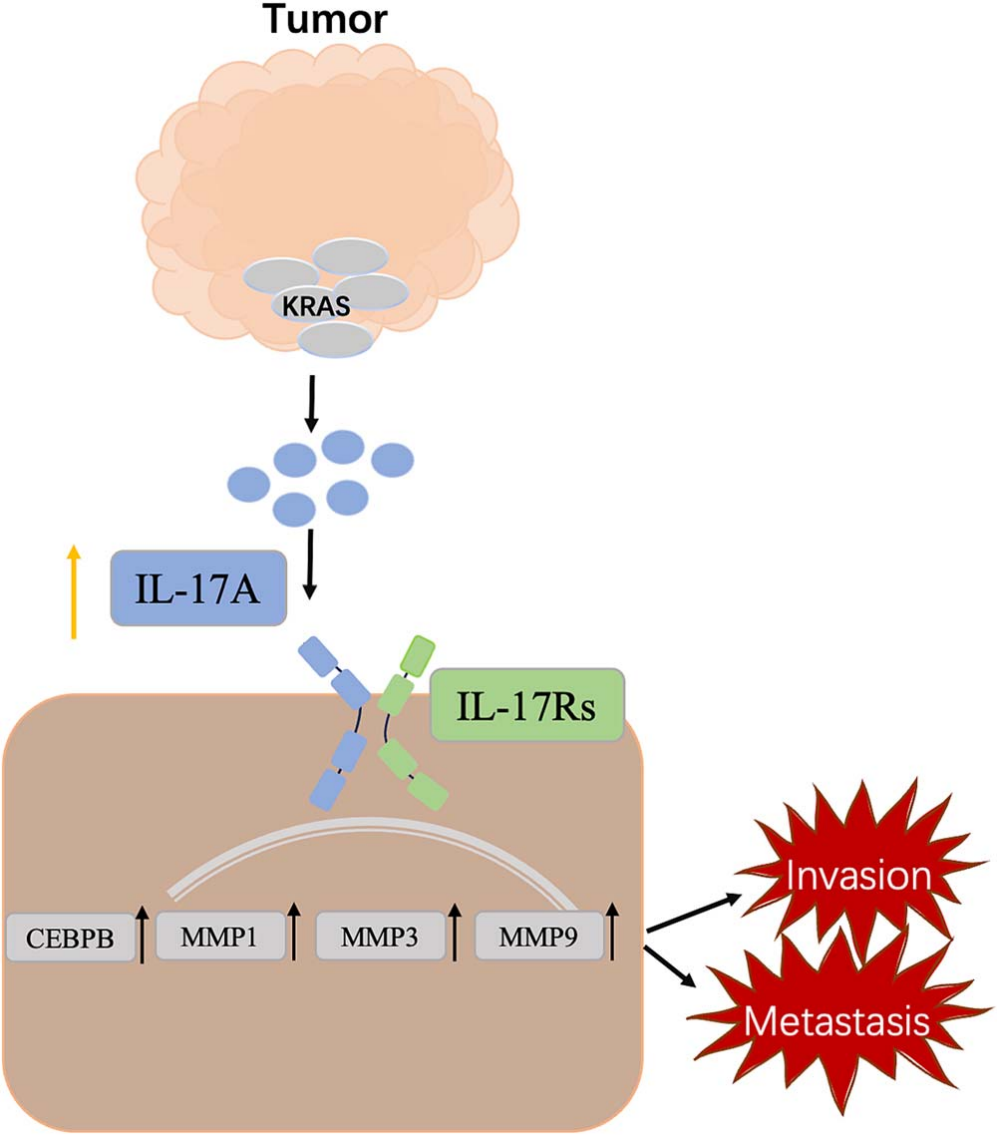
Graphical abstract. Proposed working model summarizing the mechanism of this study.

In this study, the downregulation of KRAS in human OS cells could inhibit cell migration and invasion in vitro and in vivo. Our present study demonstrates that KRAS knockdown affects tumor metastasis by inactivating IL-17 signal pathway (Figure 7). IL-17A, a crucial marker of IL-17 signal pathway, is identified as a downstream target of KRAS. Mechanistically, our data reveal that knockdown of KRAS inhibits MMP1, MMP3 and MMP9 via an IL-17 signal-dependent manner. Overall, the findings indicate that KRAS and IL-17A may serve as therapeutic targets to disrupt cooperation and reduce metastasis in human OS. The IL-17 signaling pathway mediated by KRAS could represent a universal mechanism for growth regulation that influences various other tumors, offering a new direction for future exploration. This experiment does have certain limitations. First, the sample size of the clinical study is limited because it was not a multicenter joint investigation. Second, the use of nude mice in vivo for this study may influence inflammatory infiltration. This could, in turn, impact the evaluation of IL-17A and CD45 expression levels in OS tissues. The subsequent step should involve increasing the sample size to enhance the reliability of the findings, and further research is needed to uncover the potential mechanisms involved. The next step is to expand the sample size and further validate it in humanized mice to improve the reliability of the results.

## Supplementary Material

Figure_S_ziaf056

Figure_S1_ziaf056

## Data Availability

All data in this study will be available from the corresponding author on reasonable request.
