# Correction to: KRAS regulates IL-17 signal activity by affect the metastasis of osteosarcoma via an IL-17A-dependent manner

**DOI:** 10.1093/jbmrpl/ziaf149

**Published:** 2025-10-17

This is a correction to: Xing Bao, Na Zhang, Chenchen Wang, Shiwang Liu, Aiqi Fan, Fang Zheng, Caihong Yang, KRAS regulates IL-17 signal activity by affect the metastasis of osteosarcoma via an IL-17A-dependent manner, *JBMR Plus*, Volume 9, Issue 7, July 2025, ziaf056, https://doi.org/10.1093/jbmrpl/ziaf056

In the version originally published the author Caihon Yang was incorrectly listed as affiliated with Department of Immunology, School of Basic Medicine, Tongji Medical College, Huazhong University of Science and Technology, Wuhan 430030, People's Republic of China. This has been corrected to read Department of Orthopedics, Tongji Hospital of Tongji Medical College, Huazhong University of Science and Technology, Wuhan 430030, People's Republic of China.

