# Supplementary figures and images for: KRAS regulates IL-17 signal activity by affect the metastasis of osteosarcoma via an IL-17A-dependent manner
Source: JBMR Plus. 2025 Apr 7;9(7):ziaf056. doi: 10.1093/jbmrpl/ziaf056 (PMC12143473; doi:10.1093/jbmrpl/ziaf056)

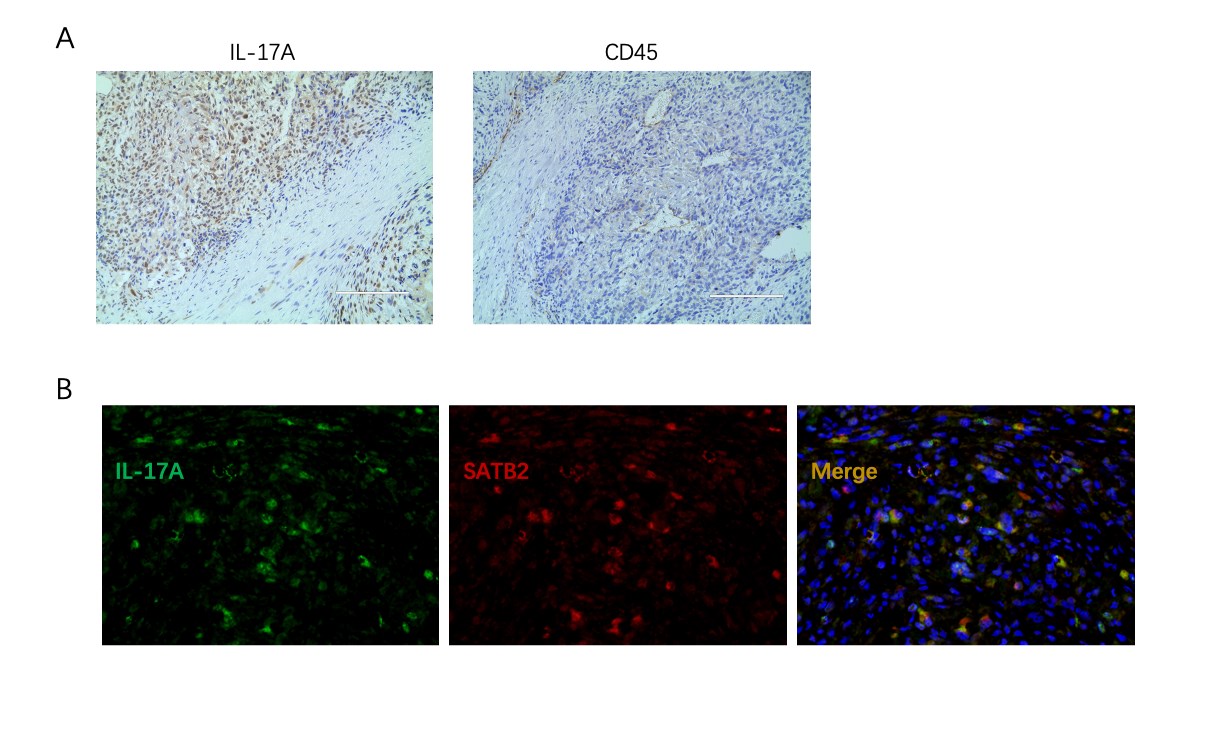

Supplement: Figure_S_ziaf056 [file figure_s_ziaf056.jpeg]
