# Supplementary material for: KRAS regulates IL-17 signal activity by affect the metastasis of osteosarcoma via an IL-17A-dependent manner
Source: JBMR Plus. 2025 Apr 7;9(7):ziaf056. doi: 10.1093/jbmrpl/ziaf056 (PMC12143473; doi:10.1093/jbmrpl/ziaf056)
Supplement: Figure_S1_ziaf056 [file figure_s1_ziaf056.docx]

Figure S1

Representative images of IL17A, SATB2 and CD45 expression in tumor cells in human osteosarcoma tissue. A. Association of CD45 and IL-17A at the invasive margin of osteosarcoma tissues by immunohistochemical staining. B. Analysis of IL-17A and SATB2 distribution in osteosarcoma tissues by immunofluorescence microscope.
